# Next‐generation metrics for monitoring genetic erosion within populations of conservation concern

**DOI:** 10.1111/eva.12564

**Published:** 2017-11-22

**Authors:** Gregoire Leroy, Emma L. Carroll, Mike W. Bruford, J. Andrew DeWoody, Allan Strand, Lisette Waits, Jinliang Wang

**Affiliations:** ^1^ Food and Agriculture Organization (FAO) of the United Nations, Animal Production and Health Division Rome Italy; ^2^ Scottish Oceans Institute and School of Biology University of St Andrews St Andrews UK; ^3^ Cardiff School of Biosciences and Sustainable Places Institute Cardiff University Cardiff UK; ^4^ Department of Forestry and Natural Resources Purdue University West Lafayette IN USA; ^5^ Department of Biological Sciences Purdue University West Lafayette IN USA; ^6^ Department of Biology Grice Marine Laboratory, College of Charleston Charleston SC USA; ^7^ Department of Fish and Wildlife Sciences University of Idaho Moscow ID USA; ^8^ Institute of Zoology Zoological Society of London London UK

**Keywords:** adaptation, conservation, effective population size, genomics, inbreeding, monitoring, single nucleotide polymorphism

## Abstract

Genetic erosion is a major threat to biodiversity because it can reduce fitness and ultimately contribute to the extinction of populations. Here, we explore the use of quantitative metrics to detect and monitor genetic erosion. Monitoring systems should not only characterize the mechanisms and drivers of genetic erosion (inbreeding, genetic drift, demographic instability, population fragmentation, introgressive hybridization, selection) but also its consequences (inbreeding and outbreeding depression, emergence of large‐effect detrimental alleles, maladaptation and loss of adaptability). Technological advances in genomics now allow the production of data the can be measured by new metrics with improved precision, increased efficiency and the potential to discriminate between neutral diversity (shaped mainly by population size and gene flow) and functional/adaptive diversity (shaped mainly by selection), allowing the assessment of management‐relevant genetic markers. The requirements of such studies in terms of sample size and marker density largely depend on the kind of population monitored, the questions to be answered and the metrics employed. We discuss prospects for the integration of this new information and metrics into conservation monitoring programmes.

## 1 INTRODUCTION

Over the last few decades, different components of biodiversity, from populations to ecosystems, have experienced a massive reduction in genetic diversity (Hughes, Inouye, Johnson, Underwood, & Vellend, 2008). In vertebrates, most threatened species have seen their genetic diversity reduced over the last few hundred years (Li et al., 2016; Willoughby et al., 2015). Most countries worldwide report significant genetic vulnerability within their plant populations: with, for example, roughly half of forest species being threatened (FAO, 2010, 2014). Furthermore, due to prolonged and intensive artificial selection, the effective population sizes of major domesticated livestock breeds rarely exceeds a few hundred individuals (Leroy et al., 2013), despite their often very large census sizes. Thus, many domestic breeds of high heritage value also need management to maintain genetic diversity (Bruford et al., 2015).

The conservation of genetic diversity is one of the priorities of the Convention of Biological Diversity (CBD; http://www.cbd.int/convention/text/). The maintenance of genetic diversity is also included in the UN's Sustainable Development Goals (https://sustainabledevelopment.un.org/). For the purpose of population monitoring, many metrics have been proposed to assess changes in genetic diversity and possible genetic erosion, including the coancestry coefficient, population allelic diversity, population differentiation and diversity of domesticated breeds and varieties (http://geobon.org/essential-biodiversity-variables/what-are-ebvs/).

Erosion usually refers to the process of gradual diminution by external forces. When dealing with biodiversity, genetic erosion refers to “the loss of genetic diversity, in a particular location and over a particular period of time, including the loss of individual genes, and the loss of particular combinations of genes”… “It is thus a function of change of genetic diversity over time.” (FAO & IPGRI, 2002, p. 3). Small or isolated populations lose genetic diversity faster than is introduced by immigration and new mutations. This loss of genetic diversity occurs through interacting mechanisms such as genetic drift or selection, exerted by various forces external to the population (Lacy, 1987). The “genetic erosion” concept was coined in a conservation/management context to denote the widespread/extreme loss of advantageous genes and genotype combinations, often driven by anthropogenic environmental change, which can drive population extinction even when census numbers and habitat appear favourable to persistence (Bijlsma & Loeschcke, 2012).

**Figure 1:**
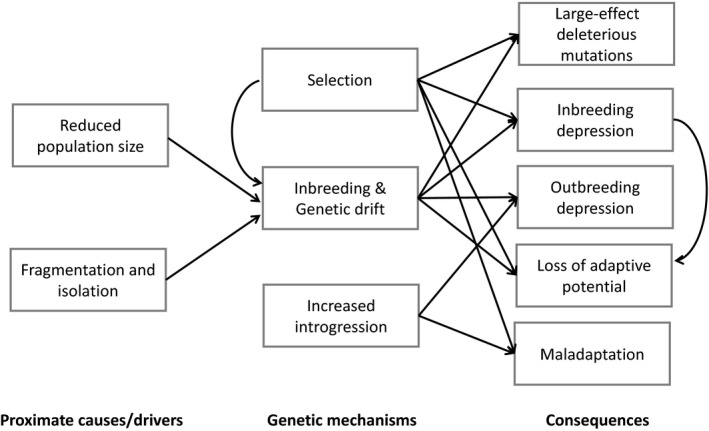
Drivers, mechanisms and consequences of genetic erosion

For effective population monitoring and management, indicators and metrics may not only be needed to infer the underlying mechanisms and external drivers of genetic erosion, but also to measure their consequences (see Figure 1). However, developments of useful indicators have been hampered, until recently, by a lack of sufficiently informative genetic markers that can be analysed efficiently and economically. However, technological advances in DNA sequencing and modern genomic approaches offer new opportunities for monitoring genetic erosion, including from a functional genetic perspective. In this context, metrics of genetic erosion need to be robust relative to the sample scheme used to characterize the population, compatible across different types of genetic marker and applicable to a wide range of species.

In this review, we evaluate genetic erosion metrics that have been developed or improved for use with high‐resolution genomic data, from the perspective of population monitoring, conservation and management, considering a wide variety of examples taken from plant and animals, wild and domesticated. We review the mechanisms, drivers and consequences of genetic erosion that can be analysed using molecular tools. Suitable metrics are discussed, profiling their potential value in population monitoring and management.

## 2 COMPONENTS OF GENETIC EROSION

For population management, assessing genetic erosion per se is an essential for monitoring evolutionary potential. It may, however, be important to differentiate the underlying processes leading to genetic erosion, such as inbreeding or genetic drift, from its proximate causes/drivers, which are the point at which management actions can have a positive impact (Figure 1). In this context, selection and introgression can be considered both as mechanisms, given their specific impact on non‐neutral diversity and drivers (e.g., artificial selection for an evolutionarily and economically important trait such as milk yield). These drivers and mechanisms may have consequences such as inbreeding and outbreeding depression, emergence of large‐effect deleterious mutations, maladaptation and the loss of adaptive potential, which can interact and amplify via feedback mechanisms and ultimately lead to extinction (Frankham, 2005). As metrics based on genomic information have been developed to monitor these consequences, they will also be investigated in this section (see Table 1). Note that we do not directly consider external drivers such as habitat loss and climate change, which influence genetic erosion but cannot be individually and separately monitored and assessed using genomic metrics.

**Table 1:** Characteristics of useful metrics for molecular monitoring of genetic erosion at the population level

Components to be monitored	Examples of metrics	Sample size required	Marker density required	Remarks
Genetic mechanisms				
Inbreeding	*F* metrics (runs of homozygosity—ROH), change in H_e_, A_e_…	Low	High	ROH: time frame adjustableHe: sensibility to ascertainment bias
Effective population size	*N*_*e*_ metrics (*N*_eI_, *N*_ev_, *N*_eLD_ …)	Low	Increasing with *N*_*e*_	*N*_eLD_: time frame adjustable
Selection	Frequency of management‐informative alleles	Low	High/low	
Introgression	Number of hybrids, % admixture…	Low	Low	
Proximate causes/drivers				
Population size and demographic parameters	*N*_*c*_, *N*_*b*_, *N*_*i*_, Φ, λ	High	Low	Long‐term monitoring can be required to gain precise estimates
Fragmentation and isolation	*F*‐statistics, Gst, *N*_*n*,_ Kinship metrics…	Low	Low	
Consequences				
Inbreeding and outbreeding depression	Heterozygosity‐fitness correlations (HFC), regression coefficients on *F* or genetic divergence	High	High	Requires specific trait phenotypic information
Emergence of large‐effect deleterious mutations	Number of loss‐of‐function (LoF) variants, frequency of management‐informative alleles	Low	High/low	
Maladaptation	Frequency of management‐informative alleles	Low	High/low	
Loss of adaptability	*V*_*a*_, *r*,* h* ^2^	High	High	Requires specific trait or phenotypic information

Low sample size and marker density are here considered to be <100 individuals and a few hundred SNPs.

One of the advantages offered by genomic tools is their ability to differentiate the impacts of genetic erosion on neutral and adaptive components of variation. Processes such as inbreeding and drift are expected to reduce genetic variation (e.g., heterozygosity) equally at both neutral and adaptive loci in small populations. However, selection acts at different levels: as a driver of genetic erosion, it will affect the number and productivity of successful breeders, thereby indirectly impacting inbreeding and drift. As a mechanism, directional selection is also expected to decrease variability at target genes and adjacent genomic regions that are linked by lack of recombination. Historically, the limitations of molecular markers available to conservation geneticists meant that it was difficult to obtain data for both marker types and evaluations of genetic diversity at neutral loci were used as a proxy for genetic variation at adaptive loci when evaluating adaptive potential and the relationship between genetic diversity and fitness (Hansson & Westerberg, 2002). This approach was shown to be effective in many studies (Keller & Waller, 2002; Reed & Frankham, 2003), but also required many neutral loci to obtain sufficient power to detect relationships (Coltman & Slate, 2003) and the correlation between neutral loci and quantitative genetic variation can be low (Reed & Frankham, 2001). Recent technical advances are now yielding datasets that do contain both (e.g., large single nucleotide polymorphisms [SNP] panels; Doyle et al., 2016). Research into domestic and captive populations has played a leading role in our understanding of how genetic variation at neutral and non‐neutral markers evolves over time (see for instance Willoughby, Ivy, Lacy, Doyle, & DeWoody, 2017). In parallel, through emerging research fields such as landscape genomics, it is becoming possible to infer gene variants driving local adaptation in the wild (Rellstab, Gugerli, Eckert, Hancock, & Holderegger, 2015).

### 2.1 Genetic mechanisms of genetic erosion

#### 2.1.1 Inbreeding, genetic drift and effective population size

Genetic drift refers to random changes in population allele frequencies due to the sampling of gametes during reproduction (Wright, 1931). Without the counteracting action of forces such as migration and mutation, genetic drift can lead to the fixation of one allele and the loss of all other alleles at a locus at a rate dependent on the effective population size (i.e., complete loss of genetic variation or fixation). The mean rate of erosion of genetic variation due to drift is expected to be the same for all neutral loci in the nuclear genome, although actual values will vary, for example, due to genetic hitchhiking (Jiménez‐Mena & Bataillon, 2016) and background selection. The level of genetic drift in a population can be monitored by estimating the variance effective population size.

Inbreeding was originally defined by Wright (1921) as the correlation between parental gametes that unite to form an individual relative to the total array of such gametes in a random sample from the reference population. Later, it was defined as the probability that two homologous genes in an individual were inherited from the same ancestral gene (identity by descent (IBD), Malécot, 1948). The application of the correlation‐based inbreeding concept to a subdivided population yields Wright's *F*‐statistics, with F_IS_ and F_IT_ being the inbreeding coefficient of an individual relative to a reference of the subpopulation and the total population, respectively.

The development of high‐density genomic data has offered opportunities to assess IBD via multilocus heterozygosity or using genomic relatedness matrices (Kardos, Luikart, & Allendorf, 2015; Willoughby et al., 2017). Another useful approach utilizes stretches of homozygosity throughout the genome (Runs of Homozygosity, ROHs), which are likely to have been inherited by descent. The history of inbreeding within a population can be estimated from the length distribution of ROH segments. This method has been viewed as one of the most promising approaches to investigate inbreeding (Bjelland, Weigel, Vukasinovic, & Nkrumah, 2013; Bruniche‐Olsen & DeWoody, [Link]; Keller, Visscher, & Goddard, 2011). It is generally considered that with high‐density data, genomic measures of inbreeding are more efficient in measuring IBD than pedigree approaches (Hoffman et al., 2014; Kardos et al., 2015). Over the last 10 years, ROH approaches have been extensively used for population analysis in livestock (Bjelland et al., 2013; Ferenčaković et al., 2013; Keller et al., 2011), and now their wild relatives (Iacolina et al., 2016; Kardos, Qvarnström, & Ellegren, 2017).

In monitoring, genetic erosion can be investigated via changes in multiple metrics of genetic diversity (e.g., heterozygosity H_e_, average coancestry, effective number of alleles A_e_, etc.; Table 1). One of the best metrics of genetic erosion is the effective population size *N*_*e*_ (Wright, 1931), that is, the size of an idealized population that would produce the same genetic variation as the population under study (Caballero, 1994; Crow & Kimura, 1970; Wang, 2016). The inbreeding effective size (*N*_eI_), which measures the rate of inbreeding (i.e., the approach to homozygosis), and variance effective size (*N*_ev_), which measures the rate of drift (i.e., the approach to fixation), are equivalent for a single population of constant size (Wang, 2005). Normally, the two metrics are different but highly correlated, except when populations fluctuate dramatically over one or a few generations or when populations are subdivided with low levels of migration. With the increasing availability of genomic data, *N*_*e*_ metrics can be estimated from various signals (such as temporal variance in allele frequency, frequency of close relatives, linkage disequilibrium; Wang, 2016). The linkage disequilibrium (LD) approach, which uses the correlation between alleles at different loci to estimate *N*_*e*_, reflects the inbreeding effective population size in the previous generation when considering unlinked loci (Hare et al., 2011), or even over a longer time‐period, when considering linked loci. This property makes it very useful in recently declining or isolated populations, and has been increasingly used in various species (Kijas et al., 2014; Makina et al., 2015; Pazmiño, Maes, Simpfendorfer, Salinas‐de‐León, & van Herwerden, 2017; Plomion et al., 2014).

Hollenbeck, Portnoy, and Gold (2016) used an extension of linkage disequilibrium to estimate *N*_*e*_ over a range of time points using SNP genotype data from a single sample per population. The method was able to detect recent changes in *N*_*e*_ without phasing of genomic data, giving it strong potential for conservation genomics. The LD approach is however not free from bias, especially due to limited population sampling or genotyping errors (Wang, 2016). More recently, methods identifying IBD tracts (equivalents to ROH) from genomic DNA sequence or SNP data have been proposed, using their length distribution to infer the *N*_*e*_ trajectories over hundreds of generations (Browning & Browning, 2015). These methods work well for historical, but not contemporary *N*_*e*_. Recently Jiménez‐Mena and Bataillon (2016) showed that genetic hitchhiking (Hill & Robertson, 1966) can render estimates *N*_*e*_ heterogenous across the genome, with a local reduction in *N*_*e*_ at neutral sites linked to adaptive regions due to the effect of background (Charlesworth, Morgan, & Charlesworth, 1993) and positive selection (Smith & Haigh, 1974).

#### 2.1.2 Selection

As an important driver of genetic erosion, selection affects genetic variation in a number of ways. Balancing selection (e.g., heterozygote advantage and frequency dependent selection) can increase locus‐specific variation, whereas directional selection can decrease it (Wright, 1984). The type and strength of selection can be detected from genetic marker data. Different approaches may be used, considering either evolution in allele frequencies, linkage disequilibrium or detection of outlier loci in population differentiation (Vitti, Grossman, & Sabeti, 2013), and in recent years, a wide number of genomic regions under selection have been detected (Cavanagh et al., 2013; Doyle et al., 2016; Gompert et al., 2014).

In genetic monitoring for conservation, the focus may be less on the detection of loci under selection and more on identifying genetic variants of interest for fitness and population persistence. For instance, in a study on a transmissible cancer affecting Tasmanian devils, Epstein et al. (2016) identified two chromosomal regions associated with immune function and cancer risk and asserted that identifying disease‐free individuals with favourable genotypes could be important for eventual future devil reintroductions. More generally, characterization of gene variants conferring an adaptive advantage may be important in genetic monitoring, as those variants may drive evolution of genetic diversity within populations.

#### 2.1.3 Introgression

Introgression refers to the flow of alleles/genes from one species into another by repeated backcrossing of interspecific hybrids with one of the parental species. It is a natural evolutionary process that can have positive impacts on biodiversity, such as an increase in genetic diversity and fitness in hybrid individuals (hybrid vigour), adaptive radiation and the creation of new species (Lewontin & Birch, 1966; Seehausen, 2004). However, it can also be a major challenge for conservation and a source of genetic erosion (Allendorf, Leary, Spruell, & Wenburg, 2001; Rhymer & Simberloff, 1996). The interbreeding of populations that were formerly isolated from each other can impair the genetic integrity of either or both populations, eventually eliminating adaptive genomic architecture when hybridization progresses to introgression, and in some instances can even lead to outbreeding depression (Frankham et al., 2011) and extinction (Allendorf et al., 2001; Rhymer & Simberloff, 1996; Wolf, Takebayashi, & Rieseberg, 2001). In contrast to selection, hybridization tends to increase neutral genetic variability, although this process is expected to be ephemeral if introgressed individuals are selected against and may ultimately lead to an overall loss of genetic diversity (Der Sarkissian et al., 2015; Lawson et al., 2017).

A number of analysis software packages are available for assessment of introgression, including Structure (Pritchard, Stephens, & Donnelly, 2000), NewHybrids (Anderson & Thompson, 2002) and Admixture (especially appropriate for SNP data; Alexander, Novembre, & Lange, 2009). Some software such as PCAdmix, which estimates local ancestry using phased data (Brisbin et al., 2012), is tailored towards genome data and may allow fine‐scale genomic dissection of such events. Although quite flexible, these approaches are based on different assumptions and hypotheses (for a review of metrics and methods, see Payseur & Rieseberg, 2016). Different metrics can be analysed, considering either the different kinds of hybrids likely to be present in the population (e.g., *F*_1_, *F*_2_ or backcrosses) or the general level of admixture in terms of the proportion of the targeted population belonging to genotype clusters identified. For example, Monzón, Kays, and Dykhuizen (2014) assessed the percentage of wolf and dog ancestries in US coyote populations based on a limited set of 63 SNPs using multidimensional scaling implemented in PLINK (Purcell et al., 2007) and STRUCTURE (see also Box 2).

When considering functional markers, modern genomic tools have allowed the identification of targeted introgression in specific genome areas (Payseur, 2010; Price et al., 2009; Wegmann et al., 2011). Barbato et al. (2017) inferred adaptive, largely unidirectional introgression of mouflon alleles into the genomes of local sheep that are involved in innate immunity and bitter taste reception by analysing 37,000 SNPs in populations in the same landscape in upland Sardinia. Similarly, von Holdt, Kays, Pollinger, and Wayne (2016) used a set of 3,102 ancestry informative SNPs to evaluate differential introgression between coyotes and grey wolves in North America and found 60 regions with differential introgression in 44 individuals. In north‐eastern coyotes, these introgressed regions were enriched for genes that affect body size and skeletal proportions.

The timing of admixture events can be more difficult to quantify, but methods have been developed to estimate this parameter (Payseur & Rieseberg, 2016). One general observation is that ancient admixture events are more likely to have shorter genomic stretches because they have been broken down by recombination. This observation is, however, invalid if admixture is remains ongoing, and in this case, the admixture profile can include a mixture of long stretches of introgression (recent events) and shorter stretches, which depending on their length can be due to ancient admixture or incomplete linage sorting (ILS) if taxa are very recently diverged. Ancient DNA analysis can be very useful to compare archetypal and introgressed genomes (Schaefer, Shapiro, & Green, 2016). For instance, Skoglund, Ersmark, Palkopoulou, and Dalén (2015) recently sequenced a 35,000‐year‐old wolf from the Taimyr peninsula in northern Russia and were able to show evidence for unidirectional introgression into Siberian and Greenland dog breeds, potentially of an adaptive nature to allow them to become functionally viable in very cold environments.

### 2.2 Proximate causes/drivers

#### 2.2.1 Changes in population size

In natural populations, levels of genetic diversity and population size are correlated, with larger populations typically harbouring the most variation and evolutionary potential (Frankham, 1996). Population declines can leave a population more susceptible to extinction in the short term due to environmental, demographic and random catastrophic events (Frankham, 1995a,b). The consequences of a decrease in population size in term of genetic stochasticity can be largely captured by *N*_*e*_ metrics. However, it is also important to assess the census population size and related metrics for monitoring demographic stochasticity (Table 1). Census size (*N*_*c*_) can be estimated using a variety of nongenetic tools, from tracking individuals using natural markings, to line‐transect studies, to counting an entire population using satellite imagery. Genetic and genomic tools can however be used to estimate census population size indirectly in a variety of ways including enumeration of the number of genotypes (Taberlet et al., 1997), classic capture recapture models (Huggins, 1989; Pollock, 1982; White & Burnham, 1999; Woodruff, Lukacs, Christianson, & Waits, 2016) and spatial and/or demographically staged capture recapture models (Carroll et al., 2013; Petit & Valiere, 2006). The advent of spatial recapture models (Efford, 2011; Royle & Young, 2008) or SNP‐based pedigree approaches (Spitzer, Norman, Schneider, & Spong, 2016) has greatly improved density estimates using genetic monitoring (Mollet, Kéry, Gardner, Pasinelli, & Royle, 2015; Russell et al., 2012; Thompson, Royle, & Garner, 2012; see for instance Box 1). Other demographic parameters such as number of breeders per cohort (*N*_*b*_), population growth, (λ), survival (Φ) or abundance (*N*_*i*_; Williams, Nichols, & Conroy, 2002) contribute important information to the monitoring of endangered species and help to identify when genetic monitoring is required (Carroll et al., 2013).

Box 1 **Use of SNPs for bear population size monitoring**

1 The monitoring of bears in northern and central Sweden was initially implemented through radio transmitters and GPS receivers. Starting in 2004, noninvasive genetic sampling was carried out in five year intervals to infer parentage and assess current population size, initially with microsatellite markers. In 2014, the Swedish province of Västerbotten switched from using microsatellites to a panel of 96 SNPs with subsequent reduction in analysis costs (Schneider, 2015). Molecular estimates of the current population size of Västerbotten using SNP‐based pedigree reconstruction (404 individuals) were found within the range of official estimates based on mark–recapture (310–459 bears; Spitzer et al., 2016). Molecular markers have been also used as a basis for assessment of bear genetic structure.

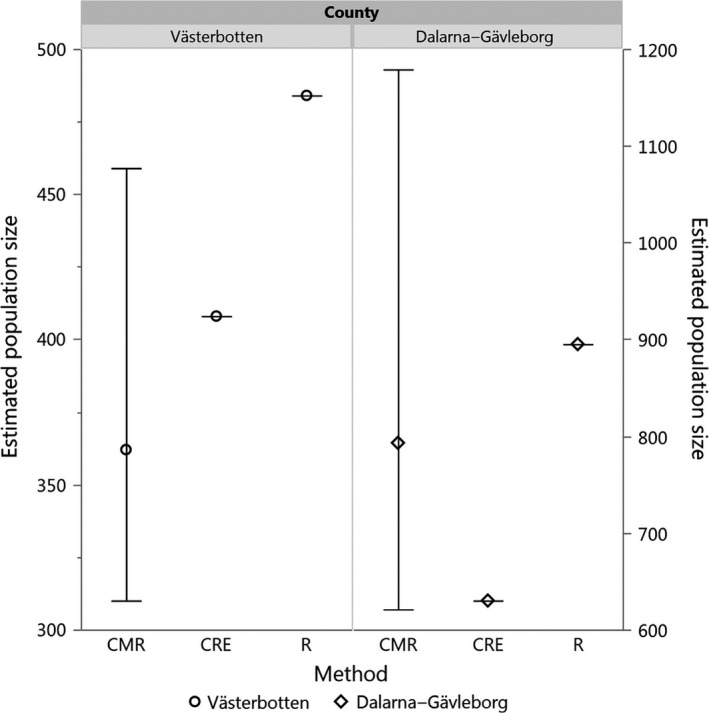

Population sizes estimates of bear population of two Swedish county based on capture–mark–recapture (CMR), Creel–Rosenblatt estimator (CRE) and rarefaction analysis (R) approaches (Spitzer et al., 2016).

Box 2 **Detecting migration trends in stock composition of sockeye salmon in real time (adapted from Dann, Habicht, Baker, & Seeb, 2013)**

1 The several dozen populations that spawn in Bristol Bay support the largest sockeye salmon (*Oncorhynchus nerka*) fishery in the world. Fluctuations in production and catches of populations are however highly variable over time, which is challenging for sustainable population‐based management. In order to detect migratory trends in stock populations, a marker set of 38 SNPs was used to perform mixed‐stock analysis and determine stock composition. Data from genetic analyses provided information on relative abundance within 4 days of capture, allowing managers to shift fishing effort among districts in anticipation of the distribution of the total return to the various stocks of origin.

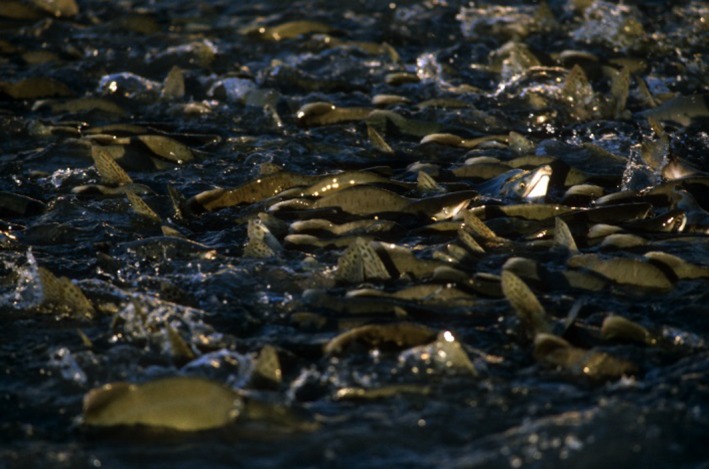

Salmon jumping in and out of the river at Katmai National Park, Alaska (©FAO/ R. Grisolia).

#### 2.2.2 Fragmentation and isolation

When a habitat becomes fragmented (e.g., by a highway or dam), the population inhabiting the region may also become fragmented, with limited migration. Over time, drift and inbreeding will increase the differentiation among populations and deplete the genetic variation in each. The differentiation between populations can be measured and monitored by *F*‐statistics (Wright, 1943) calculated from genetic marker data. However, *F*_ST_ values can take hundreds of generations to respond to the effect of new barriers to gene flow (Landguth et al., 2010), depending on factors such as *N*_*e*_ and dispersal capacity. Individual‐based genetic distance metrics such as the proportion of shared alleles are more likely to show changes over shorter timescales (10 generations), which are relevant for management (Landguth et al., 2010). Wright's neighbourhood size *N*_*n*_ can be useful when investigating genetic variation in dispersed populations (Nunney, 2016). Kinship‐based metrics that examine the spatio‐temporal distribution of related individuals (Palsbøll, Zachariah Peery, & Berube, 2010; Smouse & Peakall, 1999) have also been proposed as a useful method for detecting population structure in cases where there are low levels of genetic differentiation, such as when a barrier to gene flow is very recent. For example, Carroll et al. (2012) used paternity analysis to confirm the hypothesis that the small but significant *F*_ST_ between New Zealand and Australian right whale populations was due to reduced gene flow between them, with New Zealand males fathering calves at a rate consistent with the proportion of the population sampled, supporting the hypothesis of reduced connectivity between the two regions.

### 2.3 Consequences of genetic erosion

#### 2.3.1 Inbreeding depression

Inbreeding depression (ID) is defined as the reduction in fitness due to inbreeding, and it has been shown to affect any trait under selection (Falconer, Mackay, & Frankham, 1996; Leroy, 2014). ID metrics measure the rate at which the trait of interest changed negatively with the inbreeding coefficient (Charlesworth & Willis, 2009). A linear regression coefficient between the phenotypic value and inbreeding coefficient is the classical statistic employed (Leroy, 2014). Importantly, ID metrics are defined with reference to a specific trait and cannot be estimated solely on the basis of molecular information and require phenotypic information related to the trait of interest.

Genomic techniques have greatly enhanced the available power to assess inbreeding depression in natural or domestic populations. Such studies generally analyse heterozygosity‐fitness correlations (HFC; Hoffman et al., 2014) using a regression coefficient based on ROH (Keller et al., 2011;  Bjelland et al., 2013). ROH analysis can estimate the number of generations ago that the inbreeding occurred as well differentiating the regions involved in inbreeding depression for a given trait (see for instance Purfield, Berry, McParland, & Bradley, 2012). Finally, it is important to underline that further genomic studies are required to assess to what extent selection may have purged deleterious alleles from the genome (genetic load; Leroy, 2014).

#### 2.3.2 Outbreeding depression

Significant genomic divergence may result in complete reproductive isolation between populations, and lower levels of divergence may still reduce fitness in hybrids formed between populations (Coyne & Orr, 2004). Outbreeding depression can result from either chromosomal or genic incompatibilities between hybridizing taxa, known as intrinsic outbreeding depression, or reduced adaptation to local environmental conditions, known as extrinsic outbreeding depression (Edmands, 2007). Although reproductive isolation has been studied extensively, the effects of outbreeding depression, while widely acknowledged, have been less often demonstrated. Outbreeding depression is generally thought to be less common and less severe than inbreeding depression (Edmands, 2007; Frankham et al., 2011).

Genetic metrics for outbreeding depression should either attempt to relate the genetic divergence of alleles to fitness, requiring thus information on gametic phase, which may not always be available. As with inbreeding effects on fitness, the effects of among‐population hybridization may be difficult to predict from genetic marker variation alone; any fitness effects depend upon the differences in genetic architecture of fitness in populations, which has been shown to be highly variable among species (Edmands, 2007).

Outbreeding depression is usually determined through crossing and common‐garden experiments (Dolgin, Charlesworth, Baird, & Cutter, 2007; Edmands, 1999); approaches using next‐generation genetic data to predict outbreeding depression at the intraspecific level are currently lacking. Such studies do, however, exist at the interspecific level. For example, Christe et al. (2016) demonstrate an association between seedling fitness in early generation hybrids of *Populus alba* and *P. tremula* and the fine‐scale ancestry of chromosomal segments estimated from phased SNPs. This type of approach is likely to be successful at the species level and has the potential to help predict the importance of outbreeding depression in a conservation context (Frankham et al., 2011).

#### 2.3.3 Emergence of large‐effect deleterious mutations

The emergence of deleterious phenotypes/maladaptive traits is another consequence of genetic erosion occurring through the increase in frequency of deleterious mutations. This phenomenon can be differentiated from inbreeding depression, in that the latter is generally measured in term of its impact on a quantitative trait, whereas the former focuses on the presence or absence of a phenotype or mutation. Using whole genome resequencing, researchers can identify mutations putatively impacting the proper functioning of gene and proteins. Recently, a number of approaches, such as genomewide association studies, have been developed to identify genomic regions related to a specific trait/disorder with a limited number of examples.

More interestingly, modern genomic tools allow the identification of deleterious mutations without phenotypic information. For instance, the use of high‐density SNP panels in cattle breeds has identified haplotypes showing a deficit in homozygotes, thus enabling the detection of novel genetic defects related to prenatal deaths, allowing potential counter selection for fertility improvement (Fritz et al., 2013). In fish, Ferchaud, Laporte, Perrier, and Bernatchez (submitted) have investigated how to incorporate deleterious mutations in recommendations for management and stocking practices. A growing number of surveys of natural loss‐of‐function (LoF) variants have been carried out in vertebrates (Das, Panitz, Gregersen, Bendixen, & Holm, 2015; Groenen et al., 2012; MacArthur et al., 2012; Sulem et al., 2015; de Valles‐Ibáñez et al., 2016) and plants (Cao et al., 2011) with the number observed ranging from hundreds (332–696 in six great apes) to thousands (6,795 in Icelandic humans and ~12,000 across 80 *Arabidopsis* populations). Most recently, Rogers and Slatkin (2017) identified a much larger number of deletions retrogenes, and nonfunctioning point mutations in a woolly mammoth from Wrangel Island with a low *N*_*e*_ compared with an older sample from a larger population. This suggests that genetic erosion played a significant role in the extinction of woolly mammoths on the island and demonstrates its importance in conservation. To date, few studies have focused on estimating the fitness impacts of these variants, although Sulem et al. (2015) show that in human, homozygous LoF offspring of heterozygous parents were found in lower than expected frequencies. In *Caenorhabditis elegans*, the majority of knocked out genes reduced the fitness of the animals that carried them (Ramani et al., 2012) whereas in *Arabidopsis thaliana,* only about one‐third of knockouts had a detectable effect on fitness (Rutter, Wieckowski, Murren, & Strand, 2017). Thus, efforts to examine fitness effects of LoF mutants in model systems reveal contrasting patterns. When considering genetic monitoring and management, these approaches provide the opportunity to assess, first, the gene variant(s) behind the traits identified and the evolution of the frequency of those variants. This issue is particularly important in domestic populations, where artificial selection has resulted in accumulation of deleterious mutations (Charlier et al., 2008; Nabholz et al., 2014; Summers, Diesel, Asher, McGreevy, & Collins, 2010). Management of deleterious mutations may be integrated within selection and conservation schemes (see Box 3). For instance, the European Union implemented a genotyping and breeding programme to decrease and monitor scrapie susceptibility in sheep (including local and rare sheep breeds), which is associated with polymorphism in the prion protein gene (PRP, Brown, Orford, Tzamaloukas, Mavrogenis, & Miltiadou, 2014).

Box 3 **Managing emerging disorders in livestock: the national observatory on cattle genetic defects**

1 Increased artificial selection has caused drastic reduction in effective population size of cattle breeds and regular emergences of inherited disorders (Charlier et al., 2008). Molecular tools offer opportunities to rapidly identify causative mutations, even with a limited number of individuals genotyped. This means a potential strategy is to detect and characterize the disorders at an early stage, then provide a test that can qualify the status of the future reproducers to inform decision‐makers. In France, for instance, the national observatory on cattle genetic defects (Observatoire National des Anomalies Bovines, ONAB) is a structure that has been developed to (i) detect emergence of disorders within cattle population, through reports provided by farmers, veterinarians and technicians, (ii) gather biological and phenotypic information for further characterization of causative mutations and (iii) once carriers can be identified through dedicated gene tests, support breeding organization for the monitoring and management of the disorders (Grohs et al., 2016). This approach has allowed the identification of several causal mutations over recent years, such as the “Turning calves syndrome,” an hereditary sensorimotor polyneuropathy in Rouge des Prés breed, or an incomplete dominant neurocristopathy in Montbeliarde breed.

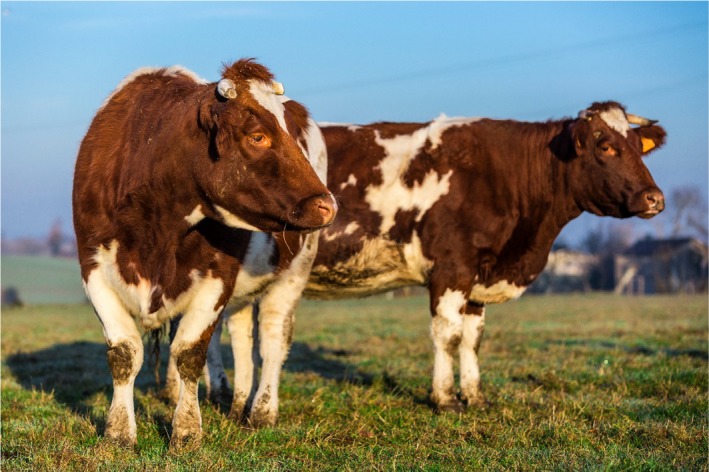

Rouge des Prés cattle in France (© SICA Rouge des prés).

#### 2.3.4 Maladaptation

We define maladaptation as the increase in deleterious phenotypes, occurring through direct or indirect effect of selection. The implication of selection differentiates maladaptation from the random emergence of deleterious phenotypes. In large populations, such traits are rare because of purifying selection. However, anthropogenic pressures can reduce population sizes to the point where genetic drift and inbreeding increase the frequency of such maladaptive phenotypes. Such human‐induced pressures include over‐exploitation, habitat destruction and artificial selection. For example, size selective harvest can lead to the evolution of smaller body sizes in fish, which leads to maladaptive traits such as fewer vertebrate, slower larval growth, high larval mortality, smaller and fewer eggs (Conover & Munch, 2002). Similar effects are seen in size‐based harvest in bighorn sheep (Coltman, O'donoghue, Jorgenson, & Hogg, 2003). Such (mal)adaptation can occur rapidly (e.g., <20 generations) in the wild and in captivity (Christie, Marine, French, & Blouin, 2012; Willoughby et al., 2017).

Genome resequencing and statistical modelling will enhance our ability to detect maladaptive genes in natural populations and, in turn, assess their frequency and impact on fitness and health. For example, Kircher et al. (2014) developed a statistical method to evaluate each variable site (i.e., SNP or indel) in the human genome and, in the light of effect sizes and genetic architecture, assign each an index of deleteriousness to prioritize whether a site may contribute to a pathogenic or maladaptive phenotype. Such measures depend, however, on a considerable volume of genomic and phenomic data, but might one day be applicable to intensively managed species of conservation concern (e.g., California condor; Romanov et al., 2009).

#### 2.3.5 Loss of adaptive potential

In comparison with the components described previously, monitoring the consequences of genetic erosion on adaptability may appear challenging. Genetic erosion can affect adaptive capacity through (i) the effects of inbreeding on phenotypic plasticity, (ii) increased magnitude of inbreeding depression under stressful conditions and (iii) reduced genetic variation for evolutionary adaptation (Bijlsma & Loeschcke, 2012; Figure 1). The two first aspects require phenotypic data, often in challenging conditions, which are rarely controlled outside the laboratory. The third aspect could in theory be monitored through the evolution of quantitative genetic parameters relating to traits of interest (such as additive genetic (co)variance, heritability *h* ^*2*^ and genetic correlation). However, a meta‐analysis by Wood, Yates, and Fraser (2016) did not find significant relationships between census population size and heritability, suggesting that adaptive potential might only be reduced at extremely small population sizes. Genomic tools facilitate the computation of these parameters, even without pedigree information, for various morphological and behavioural traits (Bérénos, Ellis, Pilkington, & Pemberton, 2014; Santure et al., 2015). As underlined by Harrisson, Pavlova, Telonis‐Scott, and Sunnucks (2014), it remains a challenge to find a robust estimator of evolutionary potential that considers all adaptive or potentially adaptive genetic (including coding, regulatory and cryptic) and epigenetic variation.

Practical genomic studies assessing the impact of genetic erosion are currently lacking. However, in the face of global environmental change, the development of methods and metrics is greatly needed to monitor adaptive potential and guide decisions from in situ or ex situ conservation to translocation (Aitken & Whitlock, 2013; Hoegh‐Guldberg et al., 2008).

## 3 MARKER SET PROPERTIES AND TEMPORAL PERSPECTIVES

Whereas hypervariable markers (such as microsatellites) were the marker of choice in the 1990s and 2000s, more recently the focus has increasingly switched to the more abundant single nucleotide polymorphisms (SNPs). For population monitoring, study requirements in terms of sampling and marker density should be carefully considered, including in relation to the time scale being considered in the analysis.

### 3.1 Precision and harmonization of metrics in relation to marker sets

In comparison with microsatellites, SNPs offer several advantages, the most important being their much higher density (Helyar et al., 2011). The density of the marker sets and individuals to be sampled should be determined by the questions under consideration (Benestan et al., 2016), as the underlying metrics, as well as parameters related to the situation of the population under study, may impact on precision (Gómez‐Romano, Villanueva, de Cara, & Fernández, 2013). Table 1 provides some general pointers for the sample size and marker density required, which would need to be adapted to the specific situation of the population under study. Characterizing most mechanisms and drivers (*N*_*e*_, census size, selection, introgression and fragmentation) in population monitoring can be accomplished using sample sizes below 100 individuals (Lenstra et al., 2012; Wang, 2016; Yates, Bernos, & Fraser, 2017). In contrast, estimates of inbreeding depression within a population require a large number of individuals (Bjelland et al., 2013; Fritz et al., 2013; Hoffman et al., 2014; Keller et al., 2011). While estimating the frequency of a given allele related to a marker of interest may require limited sampling, this number may need to be increased if the ultimate goal is to manage reproduction or introgression, requiring all potential candidates to be genotyped.

Many questions related to population size, introgression or fragmentation can be addressed with a relatively low marker density (i.e., roughly 25 microsatellites / a few hundred SNPs; Baumung, Simianer, & Hoffmann, 2004; Helyar et al., 2011); however, increasing marker density permits the characterization of more complex structure patterns (McMahon, Teeling, & Höglund, 2014). In forestry, Kramer, Degen, Blanc‐Jolivet, and Burczyk (2015) recommended that a much larger sample size per species should be sampled for population monitoring (see Box 4). For *N*_*e*_, precision is usually low for large populations (i.e., true *N*_*e*_ large) because the signal of drift is weak relative to sampling noise, requiring increased marker density (Robinson & Moyer, 2013; Wang, 2016). Other investigations focusing on functional diversity will require the use or average to high‐density marker sets, until a reduced set of management‐informative markers can be identified. For genetic monitoring with the aim of minimizing global coancestry, Gómez‐Romano et al. (2013) stated that molecular estimates outperform genealogical estimates at around 500 SNPs/Morgan. Genotype data from noninvasive or minimally invasive sampling usually have genotyping errors and missing data (Pompanon, Bonin, Bellemain, & Taberlet, 2005). Some metrics are robust to such low quality data, whereas others are not. Estimates of *F*_ST_, for example, are not strongly affected because they are calculated from allele frequencies that are robust to missing data (assuming sampling is adequate) and random mistyping. Genotype based metrics are, however, sensitive to typing errors when they are inadequately accounted for (see Carroll et al., 2017), whereas metrics based on multiple genotypes (linkage disequilibrium for instance) are sensitive to missing data.

Box 4 **Towards sustainable management of forest genetic resources (adapted from Kramer et al. 2016)**

1 With the increasing need to adapt both forest and forest management practices to climate change, several countries in Europe have developed monitoring system of their forest genetic resources. The FORGER project (towards sustainable management of forest genetic resources) aimed at providing, to actors of the European forest sector, integrated knowledge and information resources for enhanced conservation and use of forest genetic resources. Among other outputs, the project provided linkage between existing monitoring databases, like EUFGIS (http://www.eufgis.org/) and GD² (http://gd2.pierroton.inra.fr/). The project also developed a protocol for monitoring and measuring forest genetic diversity at pan‐European level. Under this protocol, monitoring should be conducted every 10 years, with at least 100 (ideally 150) adult individuals and at least 100 (ideally 150) saplings sampled per monitoring plot, for 20–50 plots per species. It was recommended to use SNPs, less prone to genotyping and scoring errors than microsatellites. The marker set should contain more than 150 SNPs, including adaptive and non‐adaptive ones. In terms of metrics of genetic diversity, it was recommended to use effective number of alleles, unordered number of genotypes, genetic distance among adults and seedlings and effective population size.

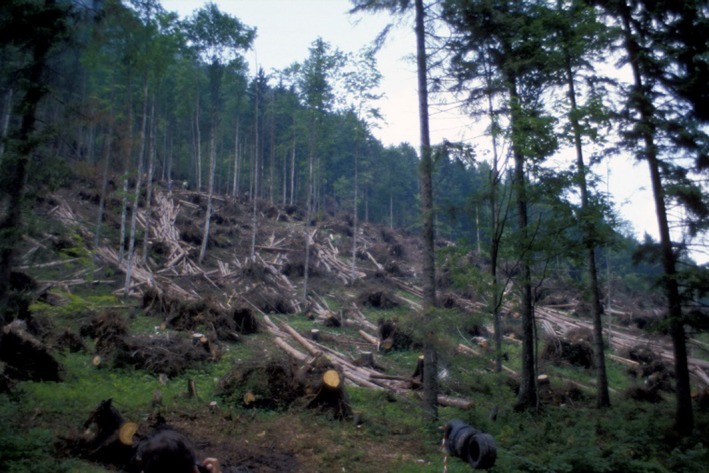

Forest uprooted by strong winds in the south of the Federal Republic of Germany (©FAO/ T. Frisk).

### 3.2 Temporal perspectives

Timescale is an important consideration for genetic metrics as (i) genetic erosion is a function of time and (ii) monitoring requires an assessment of changes occurring over different periods (Schwartz, Luikart, & Waples, 2007), with the time frames considered generally being short (one to several years/generations). Metrics to capture changes in genetic diversity over short timescales are thus particularly interesting, especially allelic diversity, which can be very sensitive to short‐term changes and in detecting demographic declines if enough SNP markers can be deployed (as few as 2500; Hoban et al., 2014).

The possibility of considering variable timescales according to physical marker linkage (N_e_LD) or marker stretch length (ROH metrics) is potentially valuable because interpolation of the historical events can be made without genotyping historic specimens. This bridges the gap when historical samples are not available and where coalescent‐based methods may be unreliable, and reduces the sampling costs involved in processing ancient or historical DNA.

However, sampling at regular time intervals should permit the monitoring of and identification of genetic erosion and its drivers, and the consequence of these factors for population fitness and health, providing insight for practitioners to take appropriate measures. As marker sets may change over time, particularly for metrics whose estimates are sensitive to the marker being used, the impact of these changes needs to be considered (through integration with former marker set or by imputation), especially when changing from microsatellite to SNPs (see Carroll et al., 2017).

## 4 USE OF INFORMATION RELATED TO SELECTED/FUNCTIONAL LOCI

As previously stated, genomic diversity may be affected differentially by genetic erosion according to whether the locus is under selection or not. Neutral markers are relatively easy to identify and can be used for many demographic analyses and metrics. Non‐neutral markers, on the other hand, are important because of their adaptive potential, their role in fitness or in any traits considered as desirable or undesirable by practitioners (DeWoody et al., 2017). Non‐neutral marker data may be of great help for the implementation of selection, translocations, reintroductions or other conservation programmes.

There are however several barriers to investigating non‐neutral genomic variation for population monitoring and as the number of steps required (read or marker filtering, phenotyping, analysis per se) is often complex, sometimes requiring advanced bioinformatics skills and resources. This can represent a serious limit to the translation of non‐neutral markers into routine management practice. Also, the necessity of phenotyping may also increase the cost of routine implementation.

Pearse (2016) has argued that for conservation decisions, an excessive focus on measurable adaptive variation could be detrimental for a population by ignoring the vast majority of functionally beneficial polymorphisms that have yet to be identified. Harrisson et al. (2014) suggested that investigations of evolutionary potential should focus on large‐effect loci when (i) both future environmental change and/or single selective pressures are known to some extent and (ii) there is good knowledge on related adaptive traits. In other cases, and especially when future changes are uncertain and/or adaptive pressures are expected to be multifaceted, genomewide variation should be used to estimate evolutionary potential. Under certain circumstances, therefore, specific traits and management‐informative genomic variants could be efficiently integrated into the monitoring of genetic erosion, if they are important for population survival and if their genomic architecture behind those traits is known. The change in frequency of such markers could be used as a complement to genomewide metrics.

## 5 USE OF GENETIC EROSION METRICS FROM THE PERSPECTIVE OF POPULATION MONITORING

Recently, there has been debate on the lack of use of genomic tools by field practitioners (Garner et al., 2016; Shafer et al., 2015). In a survey of 300 threatened species recovery plans from seven countries, Pierson et al. (2016) found that only 7% included use of molecular approaches to estimate inbreeding. In wild populations, genomic approaches are mainly used to monitor individuals (through noninvasive sampling, see Carroll et al., 2017) and populations, especially in commercial fishery species, for instance, for detecting migration trends in stock composition to inform fisheries management in real time (Box 2). However, there still seems to be a gap between the tools available and their application in the field. Even in domestic species, where dense genomic marker sets have been developed and used for breeding purposes in highly selected breeds, monitoring systems for conservation and management still rely on pedigree indicators (Verrier et al., 2015).

It is therefore important to assess to what extent genomics methods can be translated into tools useful for practitioners and decision‐makers. The examples provided in Boxes 1, 2, 3, 4, 5 provide cases studies on how metrics related to mechanisms, drivers and consequences can be integrated in monitoring programmes (see also Garner et al., 2016). Here, we discuss a number of practicalities when considering a genetic monitoring project, such as sampling regime and the use of neutral versus functional markers.

Box 5 **Monitoring genetic erosion through measuring Ne over time in the Māui dolphin**

1 The Māui dolphin (*Cephalorhynchus hectori maui*) is the subspecies of the endemic Hector's dolphin (*C. h. hectori*) that is restricted to a small segment of New Zealand's North Island (A and B). Ranked Nationally Critical under the New Zealand Threat Classification System (Baker et al., 2016), the Māui dolphin has undergone a substantial reduction in distribution and abundance since the use of nylon monofilament set nets in the late 1960s (Martien, Taylor, Slooten, & Dawson, 1999, Slooten, Fletcher, & Taylor, 2000). In order to assess population abundance and monitoring genetic erosion through estimating *Ne*, the Māui dolphin has been subject to a long‐term genetic monitoring programme. Dedicated surveys were conducted between 2001 and 2007, as well as from 2010–2011 and 2015–2016, which were augmented with the analysis of beach cast and bycaught dolphins. The figure shows the survey area and biopsy collection for 2010–2011 as an example (B), and an instance of biopsy sampling of a Māui dolphin for genetic monitoring (C). As shown by Table D, the population initially maintained a high *Nc:Ne* ratio, as expected by a population that has recently undergone a genetic bottleneck. The most recent estimate, however, shows an erosion of Ne that was expected after an initial lag period. With restrictions on set nets in place across much of the current Māui dolphin range, it is hoped that *Nc* will increase, decreasing the speed and scale of genetic erosion. B is from Hamner et al. (2014), reproduced with permission from the authors, C was provided by M. Oremus, and D is data from table 6 of Baker et al. (2016).

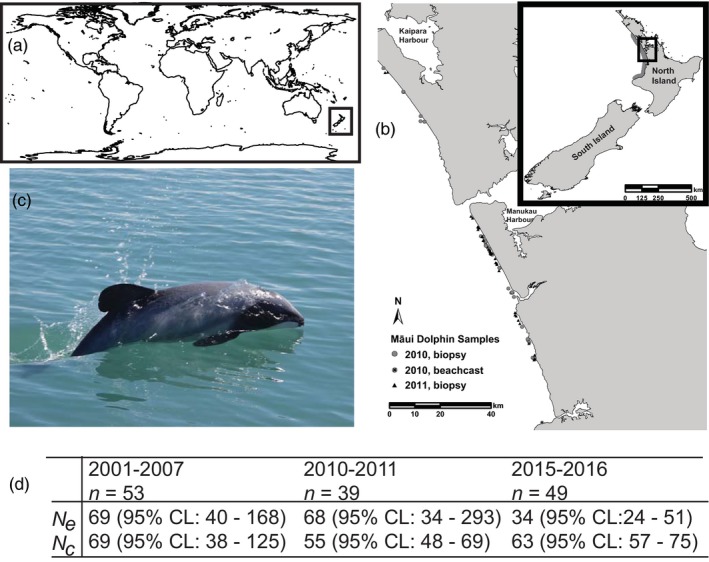

One important topic related to genetic erosion metrics relates to how those metrics can be used to trigger management interventions related to exploitation, in situ selection or conservation programmes (sampling for ex situ conservation, genetic rescue and translocation, or category‐of‐threat classification). Relative to the threats caused by climate change, Hoegh‐Guldberg et al. (2008) proposed a general decision framework to assist translocation decision. Also, Hamilton and Miller (2016) have provided the provocative suggestion that adaptive introgression may become an important future management strategy to foster climate change adaptation. Given the diversity of situations across populations, proposing general decision rules and thresholds may be difficult. However, having a clear assessment of the different components of genetic erosion may help practitioners to identify the best options. Also, monitoring of genetic parameters using genomic methods can be used to improve population viability analyses by incorporating relevant evolutionary processes (e.g., inbreeding or hybridization, Pierson et al., 2015).

**Figure 2:**
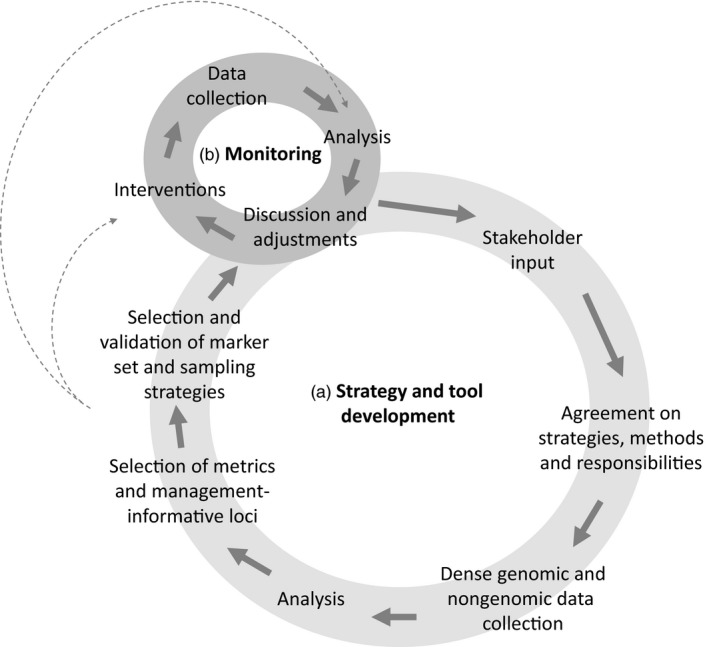
Development and use of metrics in a genetic monitoring system (adapted from Fussi et al., 2016). Dashed arrows indicate the steps in which metrics can be used

Considering how marker sets and metrics should be developed and chosen, it is important to differentiate implementation needs for genetic monitoring from the development phase, identification of loci and testing of indicators (Fussi et al., 2016). Figure 2 outlines the strategy and tool development phase (i): metrics and marker sets should be selected based on a dense marker analysis considering genomic (neutral and non‐neutral), phenotypic and environment information, allowing for the identification of management‐informative loci to address the key management questions concerning genetic erosion. Efforts should be taken to determine the minimal optimal set of data needed to efficiently yet effectively monitor genetic erosion. Once the marker set has been validated, the routine monitoring phase (ii) should utilize a low‐cost marker set (genomewide markers + management‐informative loci) and targeted low‐cost phenotypic/environment information. Both phases should be conducted as a collaborative and iterative process between managers, who will use the monitoring data, and researchers who will help design the methods and analyses. Potentially, these metrics could be usable both in the analysis and intervention steps of the monitoring cycle, for example, for exploitation/conservation interventions targeting carriers of specific alleles or management programmes aiming at minimizing molecular coancestry.

## 6 CONCLUSION

Frankham, Bradshaw, and Brook (2014) recently advocated a change in the 50/500 rule to classify conservation status of a species based on their *N*_*e*_, and Willoughby et al. (2015) made similar arguments based on *N*_*e*_ and relative levels of genetic diversity. It is not in the scope of this review to take a position on those thresholds, but our underlying arguments support the inclusion of metrics that describe the drivers and consequences of genetic erosion in population monitoring programmes.

Classical molecular tools in conservation genetics have provided useful insights into the drivers and, to some extent, the mechanisms of genetic erosion from a neutral perspective. Modern genomics approaches now offer a more complete view on the phenomenon, investigations into functional variation, as well as providing more accurate estimations of the consequences of genetic erosion on fitness and adaptation in populations. In the latter case, it is important to underline that most metrics will need to be combined with phenotypic information related to the traits relevant for fitness.

## DISCLAIMER

The views expressed in this information product are those of the authors and do not necessarily reflect the views or policies of FAO.
